# Supplementary material for: Latent endogenous giant viruses drive active infection and inheritance in a multicellular algal host
Source: Nat Microbiol. 2026 May 13;11(6):1547–58. doi: 10.1038/s41564-026-02361-z (PMC13236599; doi:10.1038/s41564-026-02361-z)
Supplement: Supplementary file 1 — Supplementary Information [file 41564_2026_2361_MOESM1_ESM.pdf]

nature microbiology

Supplementary information https://doi.org/10.1038/s41564-026-02361-z

Latent endogenous giant viruses drive active infection and inheritance in a multicellular algal host

In the format provided by the authors and unedited

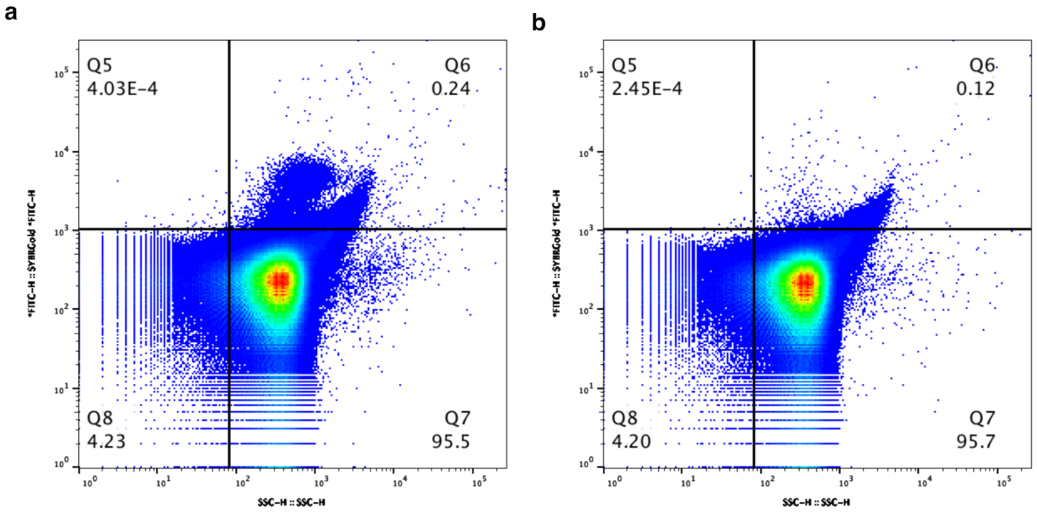

Suplementary Figure 1: Gating strategy for flow cytometry samples. Flow cytometry analysis of particles released from an alga with viral symptoms, showing a virion-like population (**a**) or from a healthy alga (**b**). Only population Q6 is presented in Figure 3 d and e, respectively.
