# Supplementary material for: Latent endogenous giant viruses drive active infection and inheritance in a multicellular algal host
Source: Nat Microbiol. 2026 May 13;11(6):1547–58. doi: 10.1038/s41564-026-02361-z (PMC13236599; doi:10.1038/s41564-026-02361-z)
Supplement: Supplementary file 2 — Reporting Summary [file 41564_2026_2361_MOESM2_ESM.pdf]

nature portfolio

Corresponding author(s): Susana Coelho

Last updated by author(s): Nov 13, 2025

Reporting Summary

Nature Portfolio wishes to improve the reproducibility of the work that we publish. This form provides structure for consistency and transparency in reporting. For further information on Nature Portfolio policies, see our Editorial Policies and the Editorial Policy Checklist.

Statistics

For all statistical analyses, confirm that the following items are present in the figure legend, table legend, main text, or Methods section.

n/a	Confirmed	
☐	☒	The exact sample size (*n*) for each experimental group/condition, given as a discrete number and unit of measurement
☐	☒	A statement on whether measurements were taken from distinct samples or whether the same sample was measured repeatedly
☐	☒	The statistical test(s) used AND whether they are one- or two-sided<br>*Only common tests should be described solely by name; describe more complex techniques in the Methods section.*
☐	☒	A description of all covariates tested
☐	☒	A description of any assumptions or corrections, such as tests of normality and adjustment for multiple comparisons
☐	☒	A full description of the statistical parameters including central tendency (e.g. means) or other basic estimates (e.g. regression coefficient) AND variation (e.g. standard deviation) or associated estimates of uncertainty (e.g. confidence intervals)
☐	☒	For null hypothesis testing, the test statistic (e.g. *F*, *t*, *r*) with confidence intervals, effect sizes, degrees of freedom and *P* value noted<br>*Give P values as exact values whenever suitable.*
☒	☐	For Bayesian analysis, information on the choice of priors and Markov chain Monte Carlo settings
☒	☐	For hierarchical and complex designs, identification of the appropriate level for tests and full reporting of outcomes
☒	☐	Estimates of effect sizes (e.g. Cohen's *d*, Pearson's *r*), indicating how they were calculated

Our web collection on statistics for biologists contains articles on many of the points above.

Software and code

Policy information about availability of computer code

Data collection	Minknown 6.8.11 for Oxford Nanopore sequencing, CFX Real-time PCR system (Biorad) for qPCR, NextSeq2000 for Illumina library sequencing (RNASeq), BD FACSMelody cell sorter for flow cytometry, JEM-2100Plus (Jeol) for TEM
Data analysis	The programs used for analysis : canu v2.2; Oxford Nanopore's Dorado, v0.6.2; Viralrecall v2.1; minimap2 v2.28; Medaka v2.0.1; Prodigal (v.2.6.3); Prodigal-gv (v.2.11.0-gv); GeneMarkS-2 (v.1.14_1.25_lic); ORForise (v.1.4.2); JBrowse2; US-align ; Orthofinder (version 2.5.5); HMMER v3.1b2 and 3.4; MAFFT v7.525 ; trimAL v1.2rev59; IQtree (v1.6.12); PyMOL v.2.5.0; AlphaFold (v.2.3.1); WebLogo3<br>Fiji (v2.14.0) for image analysis, FlowJo 10.5.3 for visualization of the flow cytometry data<br>R 4.3.0 and 4.5.1 with ggplot2 3.5.1 and DESeq2 v1.46.0 packages were used for statistical computing and graphics.

For manuscripts utilizing custom algorithms or software that are central to the research but not yet described in published literature, software must be made available to editors and reviewers. We strongly encourage code deposition in a community repository (e.g. GitHub). See the Nature Portfolio guidelines for submitting code & software for further information.

Data

Policy information about availability of data

All manuscripts must include a data availability statement. This statement should provide the following information, where applicable:

- Accession codes, unique identifiers, or web links for publicly available datasets
- A description of any restrictions on data availability
- For clinical datasets or third party data, please ensure that the statement adheres to our policy

Data is available as supplementary datasets and in the Edmond Repository https://doi.org/10.17617/3.26JRX4 . The raw sequence reads for the Oxford Nanopore data and RNA-seq libraries are available in the Sequence Read Archive under BioProject accession number PRJNA1328951.

Research involving human participants, their data, or biological material

Policy information about studies with human participants or human data. See also policy information about sex, gender (identity/presentation), and sexual orientation and race, ethnicity and racism.

Reporting on sex and gender	*Use the terms sex (biological attribute) and gender (shaped by social and cultural circumstances) carefully in order to avoid confusing both terms. Indicate if findings apply to only one sex or gender; describe whether sex and gender were considered in study design; whether sex and/or gender was determined based on self-reporting or assigned and methods used. Provide in the source data disaggregated sex and gender data, where this information has been collected, and if consent has been obtained for sharing of individual-level data; provide overall numbers in this Reporting Summary. Please state if this information has not been collected. Report sex- and gender-based analyses where performed, justify reasons for lack of sex- and gender-based analysis.*
Reporting on race, ethnicity, or other socially relevant groupings	*Please specify the socially constructed or socially relevant categorization variable(s) used in your manuscript and explain why they were used. Please note that such variables should not be used as proxies for other socially constructed/relevant variables (for example, race or ethnicity should not be used as a proxy for socioeconomic status). Provide clear definitions of the relevant terms used, how they were provided (by the participants/respondents, the researchers, or third parties), and the method(s) used to classify people into the different categories (e.g. self-report, census or administrative data, social media data, etc.) Please provide details about how you controlled for confounding variables in your analyses.*
Population characteristics	*Describe the covariate-relevant population characteristics of the human research participants (e.g. age, genotypic information, past and current diagnosis and treatment categories). If you filled out the behavioural & social sciences study design questions and have nothing to add here, write "See above."*
Recruitment	*Describe how participants were recruited. Outline any potential self-selection bias or other biases that may be present and how these are likely to impact results.*
Ethics oversight	*Identify the organization(s) that approved the study protocol.*

Note that full information on the approval of the study protocol must also be provided in the manuscript.

Field-specific reporting

Please select the one below that is the best fit for your research. If you are not sure, read the appropriate sections before making your selection.

☐ Life sciences ☐ Behavioural & social sciences ☒ Ecological, evolutionary & environmental sciences

For a reference copy of the document with all sections, see nature.com/documents/nr-reporting-summary-flat.pdf

Ecological, evolutionary & environmental sciences study design

All studies must disclose on these points even when the disclosure is negative.

Study description	Vertical transmission of viral infection in Ectocarpus sp, linked to the viral genomes insertions in the algal genomes
Research sample	Ectocarpus species 7 sporophytes showing viral symptoms or viral insertion in their genomes and available in culture collections. More details on the origin of the different algae are available in supplementary table 1
Sampling strategy	Ectocarpus species 7 sporophytes suspected to have a link with viral infection and available in culture collections were used to generate > 100 gametophyte porgeny for each sporophyte
Data collection	Each gametophyte was studied for viral symptoms and for the presence of viral insertions in its genome by microscopy and qPCR (for symptoms) and PCR (genotyping)
Timing and spatial scale	not applicable

Data exclusions	no data were excluded
Reproducibility	More than 100 individual gametophytes were generated for each sporophyte, allowing a good statistical power. Each inidividual was examined once by microscopy and PCR while the qPCR was performed in technical triplicates
Randomization	Phenotyping for viral symptoms was done in a random order with no a priori on the health status of the algae.
Blinding	Phenotyping for viral symptoms was done blindly with no a priori on the health status of the algae. Identically, PCR genotyping and qPCR was done without knowing the health stauts of the algae.

Did the study involve field work? ☐ Yes ☒ No

Reporting for specific materials, systems and methods

We require information from authors about some types of materials, experimental systems and methods used in many studies. Here, indicate whether each material, system or method listed is relevant to your study. If you are not sure if a list item applies to your research, read the appropriate section before selecting a response.

Materials & experimental systems

n/a	Involved in the study
☒	☐ Antibodies
☒	☐ Eukaryotic cell lines
☒	☐ Palaeontology and archaeology
☒	☐ Animals and other organisms
☒	☐ Clinical data
☒	☐ Dual use research of concern
☒	☐ Plants

Methods

n/a	Involved in the study
☒	☐ ChIP-seq
☐	☒ Flow cytometry
☒	☐ MRI-based neuroimaging

Plants

Seed stocks	*Report on the source of all seed stocks or other plant material used. If applicable, state the seed stock centre and catalogue number. If plant specimens were collected from the field, describe the collection location, date and sampling procedures.*
Novel plant genotypes	*Describe the methods by which all novel plant genotypes were produced. This includes those generated by transgenic approaches, gene editing, chemical/radiation-based mutagenesis and hybridization. For transgenic lines, describe the transformation method, the number of independent lines analyzed and the generation upon which experiments were performed. For gene-edited lines, describe the editor used, the endogenous sequence targeted for editing, the targeting guide RNA sequence (if applicable) and how the editor was applied.*
Authentication	*Describe any authentication procedures for each seed stock used or novel genotype generated. Describe any experiments used to assess the effect of a mutation and, where applicable, how potential secondary effects (e.g. second site T-DNA insertions, mosiacism, off-target gene editing) were examined.*

Flow Cytometry

Plots

Confirm that:

☒ The axis labels state the marker and fluorochrome used (e.g. CD4-FITC).

☒ The axis scales are clearly visible. Include numbers along axes only for bottom left plot of group (a 'group' is an analysis of identical markers).

☒ All plots are contour plots with outliers or pseudocolor plots.

☒ A numerical value for number of cells or percentage (with statistics) is provided.

Methodology

Sample preparation	0.5% gultaraldehyde fixation followed by Sybrgold staining (final concentration 4X)
Instrument	BD FACSMelody cell sorter (BD Bioscience)
Software	FlowJo 10.5.3
Cell population abundance	4 000 000 events were measured for each sample

Gating strategy

The gating strategy was used only to remove the background noise. This is more a qualitative than quantitative analysis.

☒ Tick this box to confirm that a figure exemplifying the gating strategy is provided in the Supplementary Information.
