# Supplementary figures and images for: Latent endogenous giant viruses drive active infection and inheritance in a multicellular algal host
Source: Nat Microbiol. 2026 May 13;11(6):1547–58. doi: 10.1038/s41564-026-02361-z (PMC13236599; doi:10.1038/s41564-026-02361-z)

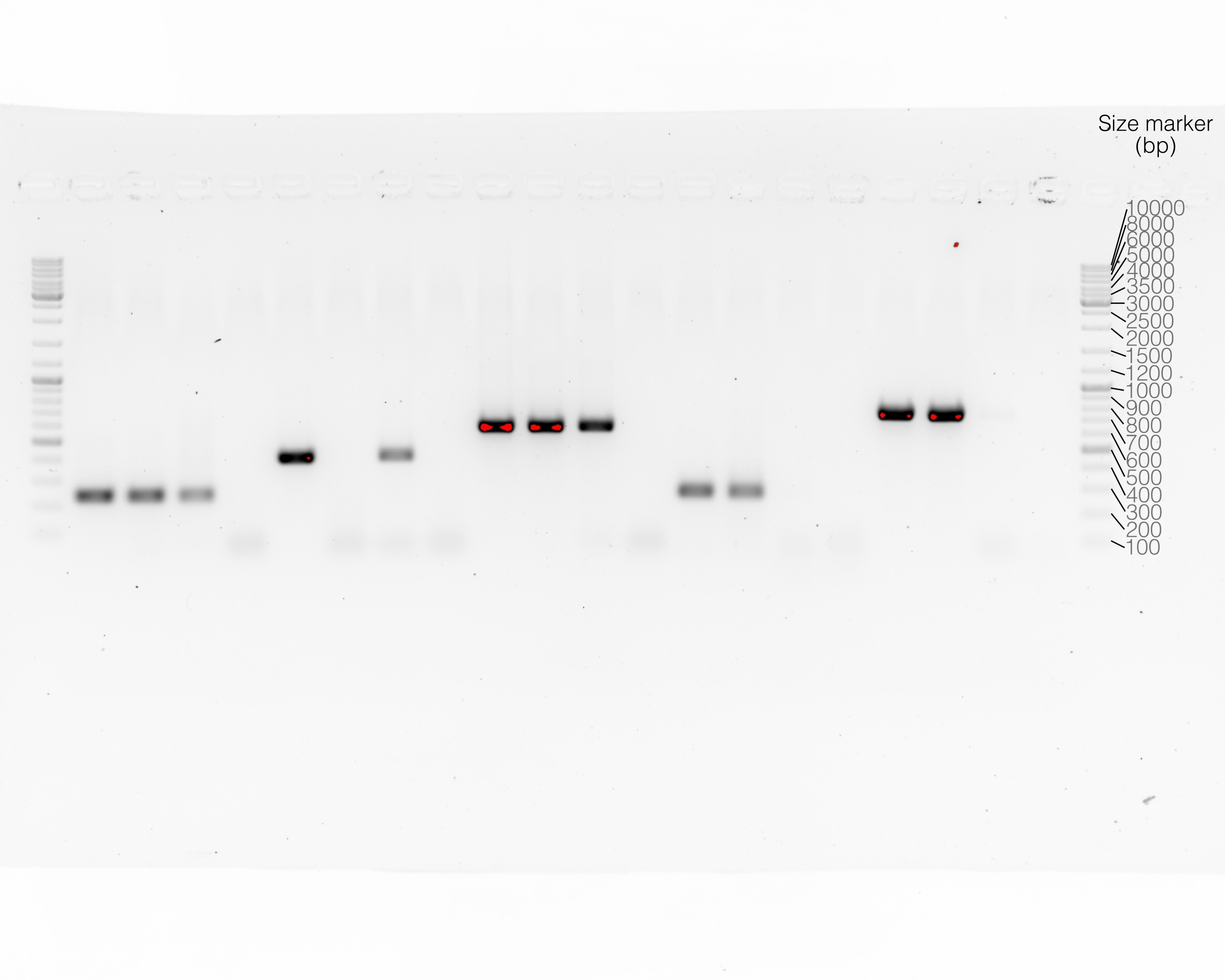

Supplement: Supplementary file 6 — Uncropped gel for Fig. 3f, with full marker size annotation. [file 41564_2026_2361_MOESM6_ESM.tiff]
